# Sensitive Electrochemical Detection of 4-Nitrophenol with PEDOT:PSS Modified Pt NPs-Embedded PPy-CB@ZnO Nanocomposites

**DOI:** 10.3390/bios12110990

**Published:** 2022-11-08

**Authors:** Mohd Faisal, Md. Mahmud Alam, Jahir Ahmed, Abdullah M. Asiri, Mohammed Jalalah, Raja Saad Alruwais, Mohammed M. Rahman, Farid A. Harraz

**Affiliations:** 1 Promising Centre for Sensors and Electronic Devices (PCSED), Advanced Materials and Nano-Research Centre, Najran University, P.O. Box 1988, Najran 11001, Saudi Arabia; 2 Department of Chemistry, Faculty of Science and Arts, Najran University, Najran 11001, Saudi Arabia; 3 Center of Excellence for Advanced Materials Research (CEAMR), King Abdulaziz University, Jeddah 21589, Saudi Arabia; 4 Department of Chemistry, Faculty of Science, King Abdulaziz University, Jeddah 21589, Saudi Arabia; 5 Department of Electrical Engineering, College of Engineering, Najran University, Najran 11001, Saudi Arabia; 6 Chemistry Department, Faculty of Science and Humanities, Shaqra University, Dawadmi 17472, Saudi Arabia; 7 Department of Chemistry, Faculty of Science and Arts at Sharurah, Najran University, Najran 11001, Saudi Arabia

**Keywords:** Pt-NPs-embedded PPy-CB/ZnO nanocomposites, 4-nitrophenol detection, differential pulse voltammetry, glassy carbon electrode, environmental safety

## Abstract

In this study, a selective 4-nitrophenol (4-NP) sensor was developed onto a glassy carbon electrode (GCE) as an electron-sensing substrate, which decorated with sol–gel, prepared Pt nanoparticles- (NPs) embedded polypyrole-carbon black (PPy-CB)/ZnO nanocomposites (NCs) using differential pulse voltammetry. Characterizations of the NCs were performed using Field Emission Scanning Electron Microscopy (FESEM), Energy-Dispersive Spectroscopy (EDS), X-ray Photoelectron Spectroscopy (XPS), Ultraviolet–visible Spectroscopy (UV–vis), Fourier Transform Infrared Spectroscopy (FTIR), High Resolution Transmission Electron Microscopy (HRTEM), and X-ray Diffraction Analysis (XRD). The GCE modified by conducting coating binders [poly(3,4-ethylenedioxythiophene) polystyrene sulfonate; PEDOT:PSS] based on Pt NPs/PPy-CB/ZnO NCs functioned as the working electrode and showed selectivity toward 4-NP in a phosphate buffer medium at pH 7.0. Our analysis of 4-NP showed the linearity from 1.5 to 40.5 µM, which was identified as the linear detection range (LDR). A current versus concentration plot was formed and showed a regression co-efficient R^2^ of 0.9917, which can be expressed by i_p_(µA) = 0.2493C(µM) + 15.694. The 4-NP sensor sensitivity was calculated using the slope of the LDR, considering the surface area of the GCE (0.0316 cm^2^). The sensitivity was calculated as 7.8892 µA µM^−1^ cm^−2^. The LOD (limit of detection) of the 4-NP was calculated as 1.25 ± 0.06 µM, which was calculated from 3xSD/σ (SD: Standard deviation of blank response; σ: Slope of the calibration curve). Limit of quantification (LOQ) is also calculated as 3.79 µM from LOQ = 10xLOD/3.3. Sensor parameters such as reproducibility, response time, and analyzing stability were outstanding. Therefore, this novel approach can be broadly used to safely fabricate selective 4-NP sensors based on nanoparticle-decorated nanocomposite materials in environmental measurement.

In this study, a selective 4-nitrophenol (4-NP) sensor was developed onto a glassy carbon electrode (GCE) as an electron-sensing substrate, which decorated with sol–gel, prepared Pt nanoparticles- (NPs) embedded polypyrole-carbon black (PPy-CB)/ZnO nanocomposites (NCs) using differential pulse voltammetry. Characterizations of the NCs were performed using Field Emission Scanning Electron Microscopy (FESEM), Energy-Dispersive Spectroscopy (EDS), X-ray Photoelectron Spectroscopy (XPS), Ultraviolet–visible Spectroscopy (UV–vis), Fourier Transform Infrared Spectroscopy (FTIR), High Resolution Transmission Electron Microscopy (HRTEM), and X-ray Diffraction Analysis (XRD). The GCE modified by conducting coating binders [poly(3,4-ethylenedioxythiophene) polystyrene sulfonate; PEDOT:PSS] based on Pt NPs/PPy-CB/ZnO NCs functioned as the working electrode and showed selectivity toward 4-NP in a phosphate buffer medium at pH 7.0. Our analysis of 4-NP showed the linearity from 1.5 to 40.5 µM, which was identified as the linear detection range (LDR). A current versus concentration plot was formed and showed a regression co-efficient R^2^ of 0.9917, which can be expressed by i_p_(µA) = 0.2493C(µM) + 15.694. The 4-NP sensor sensitivity was calculated using the slope of the LDR, considering the surface area of the GCE (0.0316 cm^2^). The sensitivity was calculated as 7.8892 µA µM^−1^ cm^−2^. The LOD (limit of detection) of the 4-NP was calculated as 1.25 ± 0.06 µM, which was calculated from 3xSD/σ (SD: Standard deviation of blank response; σ: Slope of the calibration curve). Limit of quantification (LOQ) is also calculated as 3.79 µM from LOQ = 10xLOD/3.3. Sensor parameters such as reproducibility, response time, and analyzing stability were outstanding. Therefore, this novel approach can be broadly used to safely fabricate selective 4-NP sensors based on nanoparticle-decorated nanocomposite materials in environmental measurement.

## 1. Introduction

Generally, 4-nitrophenol (4-NP), also known as para-nitro-phenol, is an organic pollutant. It is acidic in nature and moderately soluble in water. Due to its industrial importance, it has diversified industrial, pharmaceutical, and agricultural applications and is used in medicine, dyes, insecticides, herbicides, and fungicides [1]. For widespread industrial activities, thousands of tons of industrial waste are released into the environment, which has hazardous impacts on soils and ground and underground water. Subsequently, there is a great possibility of transferring it into human and animal bodies through the food chain [2]. The numerous physiological syndromes such as cyanosis, nausea, drowsiness, and headaches may appear in humans by the acute ingestion or inhalation of 4-NP. Contact of 4-NP with human eyes and skin may cause irritation [3,4]. Based on the toxicity of 4-NP, long-term exposure can cause irreversible damage to the liver, kidneys, and nervous system [5,6]. Therefore, 0.34 mg/cm^3^ 4-NP is considered the upper limit in potable water [7]. Considering the hazardous effects of 4-NP, it is important to detect and quantify various water sources in soil and the environment. Detection techniques such as gas chromatography (GC–MS), surface-enhanced Raman spectroscopy (SERS), liquid chromatography (LC) coupled with mass spectrometry (LC–MS), capillary electrophoresis, fluorescence, and high-performance liquid chromatography (HPLC) are widely used for these purposes [8]. These technologies are limited by their costly equipment, operating complications, and need for special arrangements and facilities. Therefore, these methods are suitable for clinical diagnosis but not for outdoor analysis. Thus, we propose a cost-effective and portable measuring system which is easy to use and necessary for the in situ diagnosis of the environmental unfriendly 4-NP.Electrochemical methods, including cyclic voltammetry (CV), differential-pulse voltammetry (DPV), linear-sweep voltammetry (LSV), and current–voltage (I-V) relation, are extensively utilized by researchers to develop potential detecting chemicals and measuring systems or devices. Due to the poor electron transfer rate on the working electrode of these methods, good outcomes are not obtained in this regard. Therefore, the working electrode surfaces in these methods are modified by coating them with sensing substrates (such as metal oxide nanoparticles or organometallic nanocomposites) to obtain enhanced performance [9,10,11,12]. Thus, the aim of this study is to develop an electrochemical sensor applying differential pulse voltammetry (DPV) to analyze 4-NP in a conductive aqueous medium. Metal oxides such as Cu_2_O NPs [13], ZnBi_38_O_60_ nano-bundles [14], doped metal oxide nanocomposites [15], and ZnO-Au NPs [10] have been used to modify the working electrode of the 4-NP sensor via the DPV method. On the other hand, various organometallic nanocomposites such as GCEs modified by graphene-chitosan (GR-CS) [16] and Ag NP-chitosan composites [17], have been studied for the detection of 4-NP by applying the DPV method. In addition, poly(methyl orange) has also been studied to analyze 4-NP [18]. Therefore, polymer nanocomposites decorated with metal oxides have been applied to detect 4-NP in an aqueous phase.

In this study, a polymer (polypyrole) was composited with carbon black and doped with ZnO. Further, Pt^4+^ was dispersed in the resulting polymer composites to obtain Pt NP-coated PPy-CB@ZnO NCs and was used as an electron-sensing substrate towards 4-NP in a phosphate buffer medium. Polypyrole composites such as C-dots/PPy [19], PPy–CS–Fe_3_O_4_/ITO NCs [20], and PPy/C-doped ZnO NCs [21] have been used as potential sensing materials for picric acid, glucose, and hydroquinone, respectively. On the other hand, mesoporous Si/PPy NCs [22], Ag-rGO NSs [23], and FeOx/TiO_2_@C NCs [24] have shown a higher sensitivity with wider detection ranges of 1 × 10^−8^~1 × 10^−2^ M, 2.0~150.0 mM, and 5.0–310.0 μM, respectively, in the detection of 4-NP. Therefore, the electrochemical recognition of 4-NP using a GCE with Pt NP-embedded PPy-CB/ZnO NCs was achieved to enhance its parameters, such as sensitivity, LDR, LOD, stability, response time, and reproducibility. 

## 2. Materials and Method

### 2.1. Chemical Reagents

Here, zinc acetate, Pluronic-F127 copolymer, polypyrrole-incorporated carbon black (CB), poly(3,4-ethylenedioxythiophene) polystyrene sulfonate (PEDOT:PSS), hexachloroplatinic (IV) acid hydrate, monosodium phosphate, disodium phosphate, acetic acid, hydrochloric acid, and ethanol (C_2_H_5_OH) were purchased from Sigma-Aldrich (St. Louis, MI, USA). These chemicals were used without further treatment. In the first stage, the sol–gel method was utilized for the preparation of semiconductor metal oxide (ZnO), as per previous published work [25]. Shortly, 1.6 g of Pluronic F127 was dissolved in 30.0 mL of ethyl-alcohol with constant stirring for an hour. In this solution, 2.4 g of zinc acetate, 2.3 mL of CH_3_COOH, and 0.74 mL of HCl were added and stirred for 60 min to prepare a homogeneous mixture. The solution was then left for 2 days at room temperature for proper polymerization and gel formation. The prepared mixture was then placed in a humidity chamber for aging, and finally dried at 65 °C overnight. The as-synthesized nanomaterial was then annealed at 450.0 °C for 4 h (heating at 1 °C/min and cooling at 1.0 °C/min to finally obtain the semiconductor metal oxide).

In the next step, an ultra-sonication route was used for the synthesis of 10% PPy-CB@ZnO NCs. Usually, 2.0 g of the prepared ZnO and 0.2 g of PPy-CB were stirred in 100.0 mL of double-distilled water for 15 min, followed by ultra-sonication for 30 min. The obtained mixture was properly filtered and washed (4–5 times) with ethanol and distilled water, before drying in an oven (65.0 °C/24 h) to achieve 10% PPy-CB@ZnO. Here, Pt^4+^ ions were well-dispersed into PPy-CB@ZnO composites utilizing a photo-chemical reduction approach to design Pt NP-decorated PPy-CB@ZnO NCs. Briefly, a required concentration of an H_2_PtCl_6_ solution in water containing 1.0 wt % Pt was added to a 1.0 g solution of 10% PPy-CB@ZnO in 100.0 mL of water. The suspending solution was irradiated for 14 h with an Osram Hg lamp with an illumination intensity of 2.0 mW/cm. Afterwards, filtration, washing, and drying in an oven at 65.0 °C for 24 h was performed to obtain 1% Pt-NP-embedded 10% PPy-CB@ZnO, designated as Pt-NP-embedded PPy-CB@ZnO NCs throughout this research article.

### 2.2. Materials Characterization

For the characterization of Pt-NPs-embedded PPy-CB@ZnO NCs, powder XRD investigations were conducted using a Bruker AXS-D4 Endeavour-X diffractometer (Radiation sources, CuKα_1/2;_ λα_1_ = 154.060 pm; λα_2_ = 154.439 pm). Morphological and surface-associated characterizations were made using a field-emission secondary electron microscope (FE-SEM) (JEOL-6300F, 5.0 kV; Tokyo, Japan) and a high-resolution transmission electron microscope (HR-TEM) (JEOL JEM-2100F-UHR; Tokyo, Japan), respectively. These microscopes are operated at 200.0 kV with a built-in 1K-CCD camera (Tokyo, Japan) and a Gatan GIF 2001 energy filter (Tokyo, Japan). For functional group characterization, FTIR analysis of KBr pellets in dispersion mode was performed with a Perkin Elmer Raman Station 400 spectrometer (Waltham, MA, USA) (range from 400 to 4000 cm^−1^). A VGESCALAB 200R spectrometer (Waltham, MA, USA) fitted with an MgKα (hν = 1253.6 eV) non-monochromatic X-ray source and a hemispherical electron analyzer was used to perform X-ray photoelectron spectroscopy (XPS) analysis. During the initial treatment process, freshly prepared samples were degassed, before they were shuffled in an ultra-high vacuum analysis chamber for 1 h. A Quantachrome NOVA 4200 analyzer (Florida, USA) was used at a temperature of 77.0 K to acquire the nitrogen adsorption isotherm for surface analysis. Each sample was degassed at 200.0 °C overnight. The nanocomposite surface area was measured via the Brunauer–Emmett–Teller (BET) technique using adsorption data. Diffuse reflectance spectra in a range from 200 to 800 nm were obtained on a UV–Vis spectrophotometer (Shimadzu: UV-3600 plus, Tokyo, Japan).

### 2.3. Fabrication of a GCE Using Pt NP-Embedded PPy-CB@ZnO NCs

**Scheme 1:**
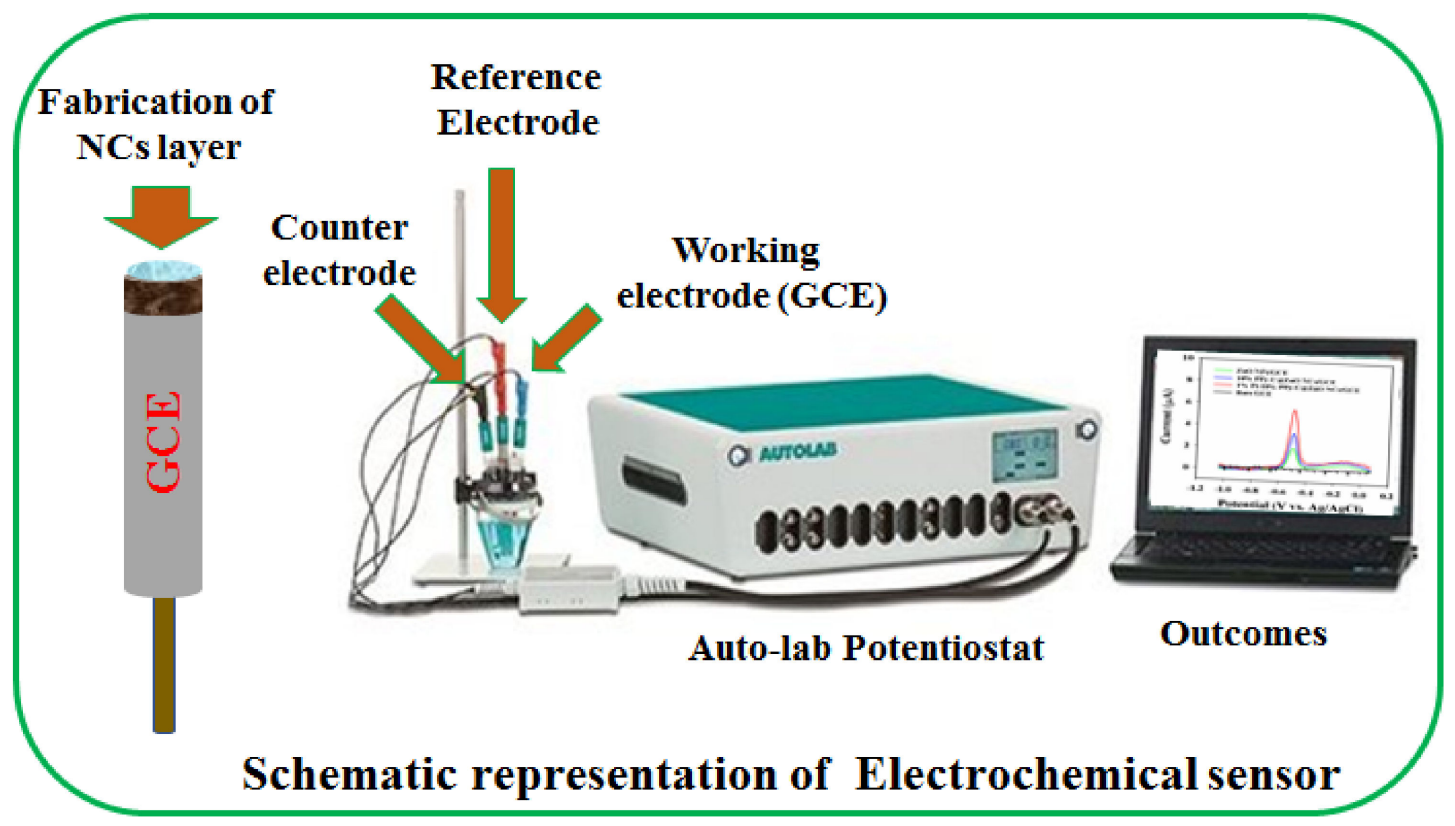
Modification of GCE with Pt-NPs-decorated PPy-CB@ZnO NCs using PEDOT:PSS binder.

Modification of GCE to a working electrode is a critical task in the sensor development. To do this, the prepared NCs were used to make slurry in ethanol, and they were used to coat the GCE surface in a way that yielded a uniform thin layer of NCs on the GCE (Surface area 0.0316 cm^2^). 10.0 µL of PEDOT:PSS were added to serve as the polymer mixture on the GCE. After drying, the modified GCE, acting as a working electrode, was supplemented with Metrohm-Autolab modules (Malden, MA, USA) at a parallel connection with an Ag/AgCl column electrode and a Pt wire (used as reference) and counter electrodes, respectively. The target analyte (4-NP) was analyzed electrochemically at a concentration range of 1.5~40.5 µM. The obtained data (e.g., sensitivity, response time, LOD, LOQ, reproducibility, and LDR) were evaluated. Finally, the real samples contaminated with 4-NP were measured. Scheme 1 represents the sensor fabrication and settings with the potentiostat.

## 3. Results and Discussion

### 3.1. Morphological Analysis of Pt NP-Embedded PPy-CB@ZnO NCs 

**Figure 1:**
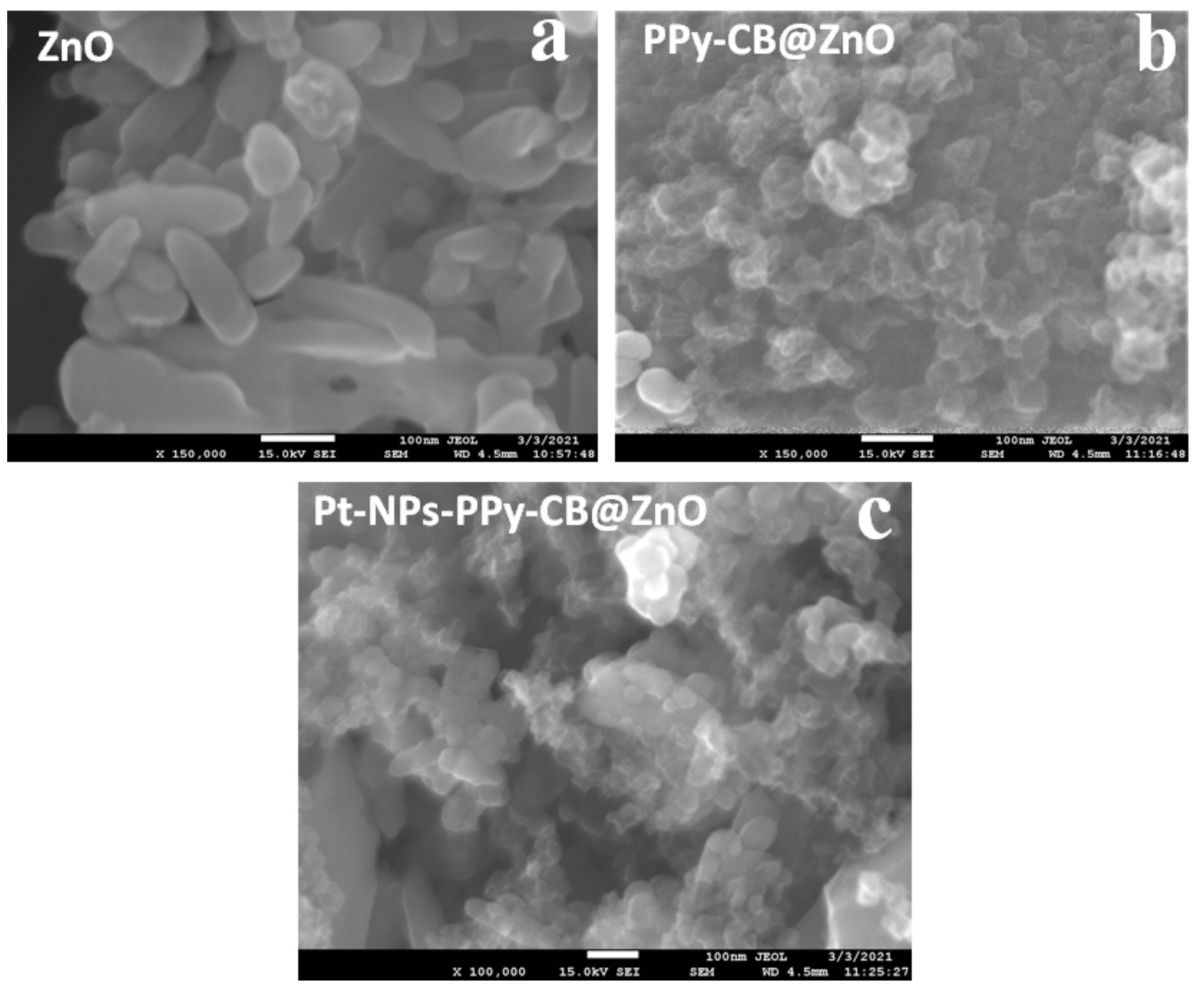
The FESEM analysis of Pt NPs-embedded PPy-CB@ZnO NCs explored (**a**) the capsule-like structure of ZnO, (**b**) the image of PPy-CB@ZnO, and (**c**) the nanocomposite structure of prepared Pt NPs-embedded PPy-CB@ZnO.

Figure 1 represents the FESEM analysis of the Pt NP-embedded PPy-CB@ZnO NCs. As presented in Figure 1a, the shape of the pure ZnO exhibits a capsule-like structure. When it was composited with polypyrrole (PPy) and carbon black (CB), the prepared ZnO particles were adsorbed onto the surface and formed a shape without any particular structure, as demonstrated in Figure 1b. After the addition of Pt nanoparticles by photo-reduction, they were also deposited onto the surface of the PPy-CB@ZnO NCs, resulting in a spherical shape. Thus, Pt NP-embedded PPy-CB@ZnO is considered a nanocomposite (NC). 

**Figure 2:**
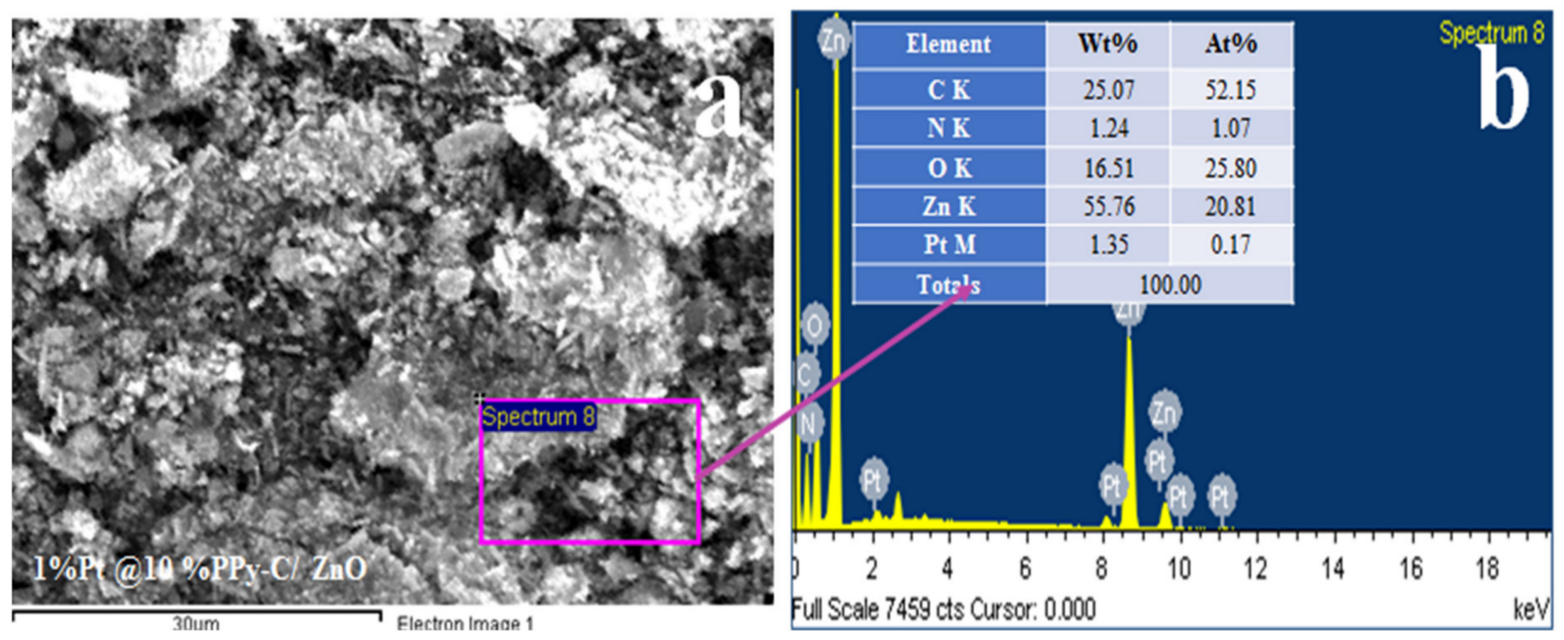
EDS analysis of Pt NPs- embedded PPy-CB@ZnO NCs; (**a**) Higher magnifying image of Pt-NPs PPy-CB@ZnO NCs and (**b**) Elemental composition spectrum of synthesized Pt NPs- embedded PPy-CB@ZnO NCs (Composition of elements; Inset; Table).

Analogous to Figure 1, a highly magnified EDS image exhibited nanocomposite-shape, as shown in Figure 2a. The elemental analysis obtained by EDS (selected area) confirms the existence of 25.07% C, 1.24% N, 16.51% O, 55.76% Zn, and 1.35% Pt, as presented in Figure 2b. Therefore, EDS analysis provides evidence of the existence of C, N, O, Zn, and Pt in the prepared Pt NPs-embedded PPy-CB@ZnO NCs. 

**Figure 3:**
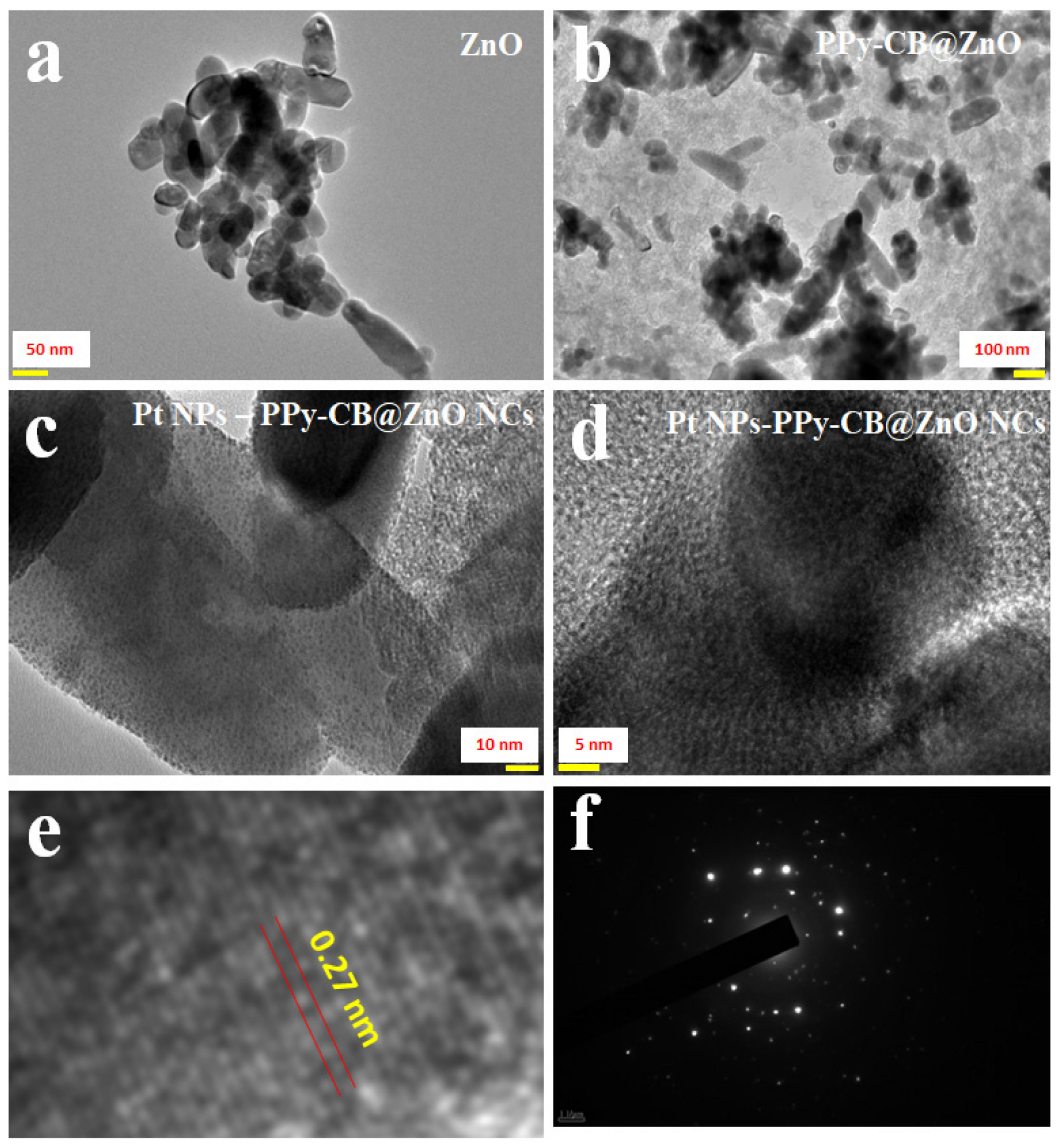
HRTEM analysis of Pt NPs-embedded PPy-CB@ZnO NCs; (**a**) ZnO, (**b**) PPy-CB@ZnO, (**c**,**d**) Low-to-high magnified Pt NPs-embedded PPy-CB@ZnO NCs, (**e**) Lattice spacing of NCs, and (**f**) diffraction pattern (SAED) of NCs.

For a detailed morphological analysis of Pt-NP-decorated PPy-CB@ZnO NCs, HRTEM was employed to confirm their structure. The obtained images are presented in Figure 3. Figure 3a shows the capsule structure of ZnO. Similarly, Figure 3b–d show the irregular adsorption of ZnO on the surface of PPy-CB and subsequently, Pt-NPs on PPy-CB@ZnO, respectively, not forming any particular shape. From the magnified HRTEM images, the lattice spacing (Figure 3e) was calculated as 0.27 nm, and the SAED is given in Figure 3f.

**Figure 4:**
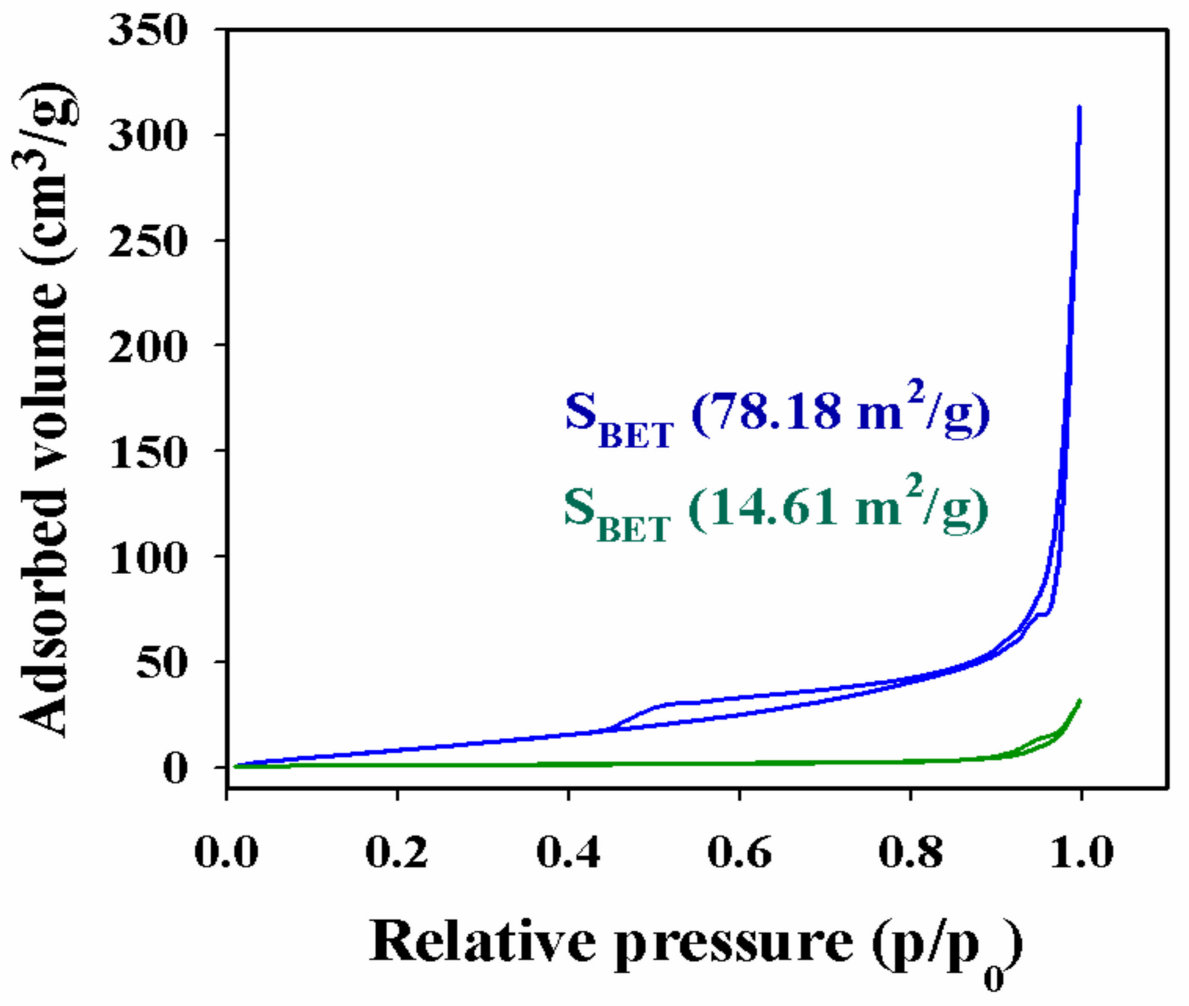
The BET analysis of Pt-NPs-embedded PPy-CB@ZnO NCs (blue line) and ZnO (green line).

The active surface area was measured for the Pt-NP-embedded PPy-CB@ZnO NC materials and is presented in Figure 4. Here, the characteristics of the prepared NCs are clarified through a nitrogen adsorption/desorption isotherm, known as BET analysis. A plot of the relative pressure versus the adsorption of nitrogen gas was used to calculate the relative surface area of NCs found as 78.18 m^2^/g. The surface area is significantly improved for the NCs (blue line; 78.18 m^2^/g) compared to only ZnO (green line; 14.61 m^2^/g) due to the increasing the functional active site by different components in the NCs. Therefore, the morphological studies of the NCs showed the surface area value is favorable to electro-catalytic performance of the fabricated sensor probe. 

### 3.2. Surface Composition by XPS Analysis

**Figure 5:**
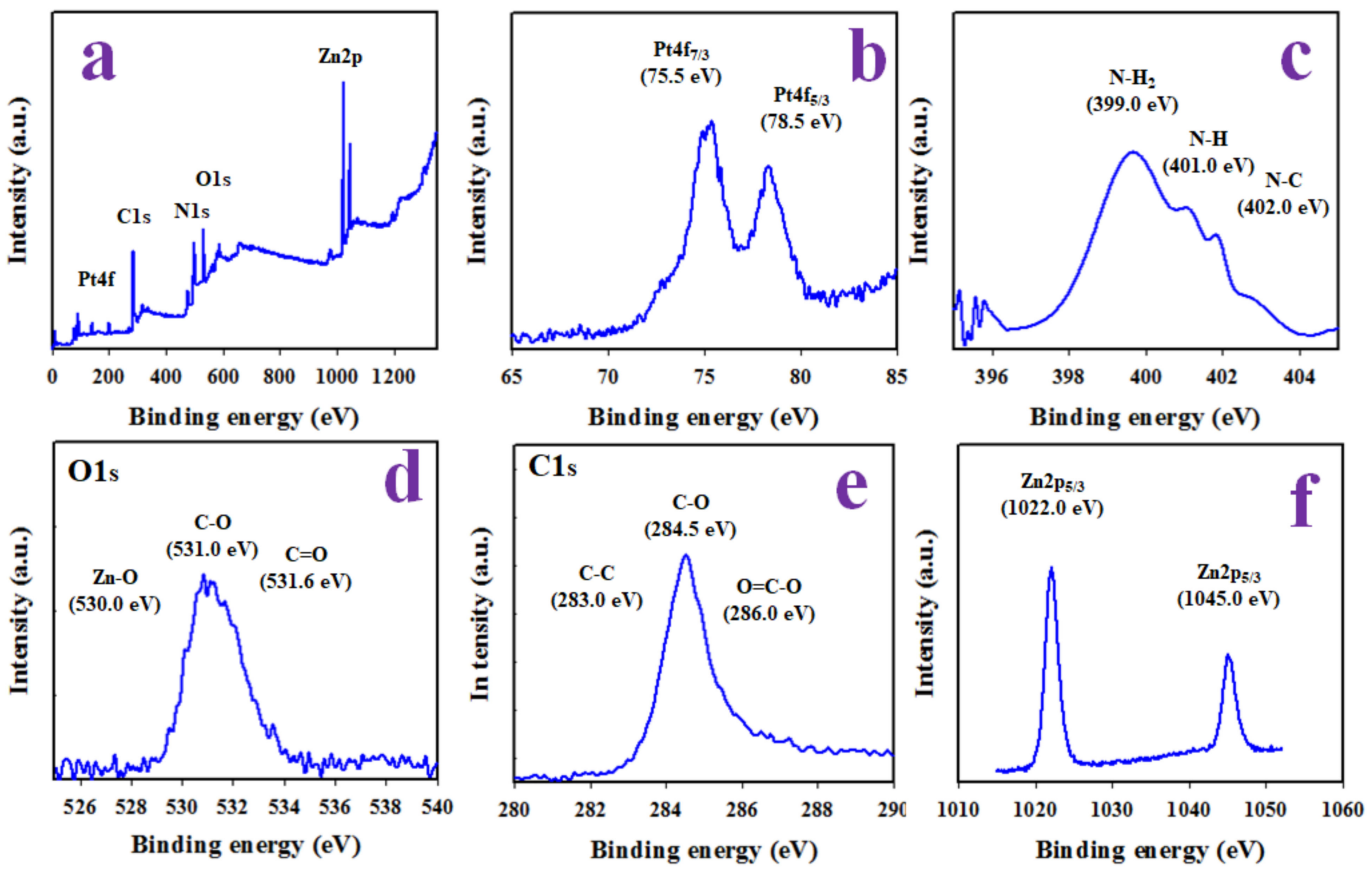
XPS analysis of Pt-NPs-embedded PPy-CB@ZnO NCs; (**a**) survey spectrum, (**b**) Pt4f, (**c**) N1s, (**d**) O1s, (**e**) C1s, and (**f**) Zn2p.

XPS analysis of the Pt-NP- embedded PPy-CB@ZnO NCs was performed to identify the oxidation states of the individual constituting atoms, as demonstrated in Figure 5. The survey spectrum of NCs has been analyzed by XPS, and it is presented in Figure 5a. The XPS spectrum of Pt4f_7/2_ and Pt4f_5/2_ is fitted with the binding energies of 75.5 and 78.5 eV, respectively, as shown in Figure 5b, which has been found for Pt^4+^ ionization, as reported elsewhere [26,27]. Nitrogen is a constituting element of Pt-NP-embedded PPy-CB@ZnO NCs, and the N1s orbital is explored in Figure 5c. As shown, the three bonds of nitrogen are positioned at 399, 401, and 402 eV, corresponding to N-H_2_, N-H, and N-C, respectively [28,29]. O1s also shows three bonds, Zn-O, C-O, and C=O, placed at 530, 531, and 531.6 eV, respectively, and confirmed the oxygen (II) oxidation state illustrated in Figure 5d [30]. Carbon is another constituting element of NCs, and the C1s orbital is illustrated in Figure 5e and contains C-C, C-O, and O=C-O bonds, which are located at 283, 284.5, and 286 eV, in accordance with results reported elsewhere [31,32]. As displayed in Figure 5f, the Zn2p orbital exhibits two-spin orbitals Zn2p_5/2_ and Zn2p_3/2_, positioned at 1022 and 1045 eV, respectively. The 23.0 eV energy difference between these two spin orbitals confirmed the existence of the Zn^2+^ oxidation state in the prepared NCs [33,34].

### 3.3. Structural and Optical Characterization of NCs

**Figure 6:**
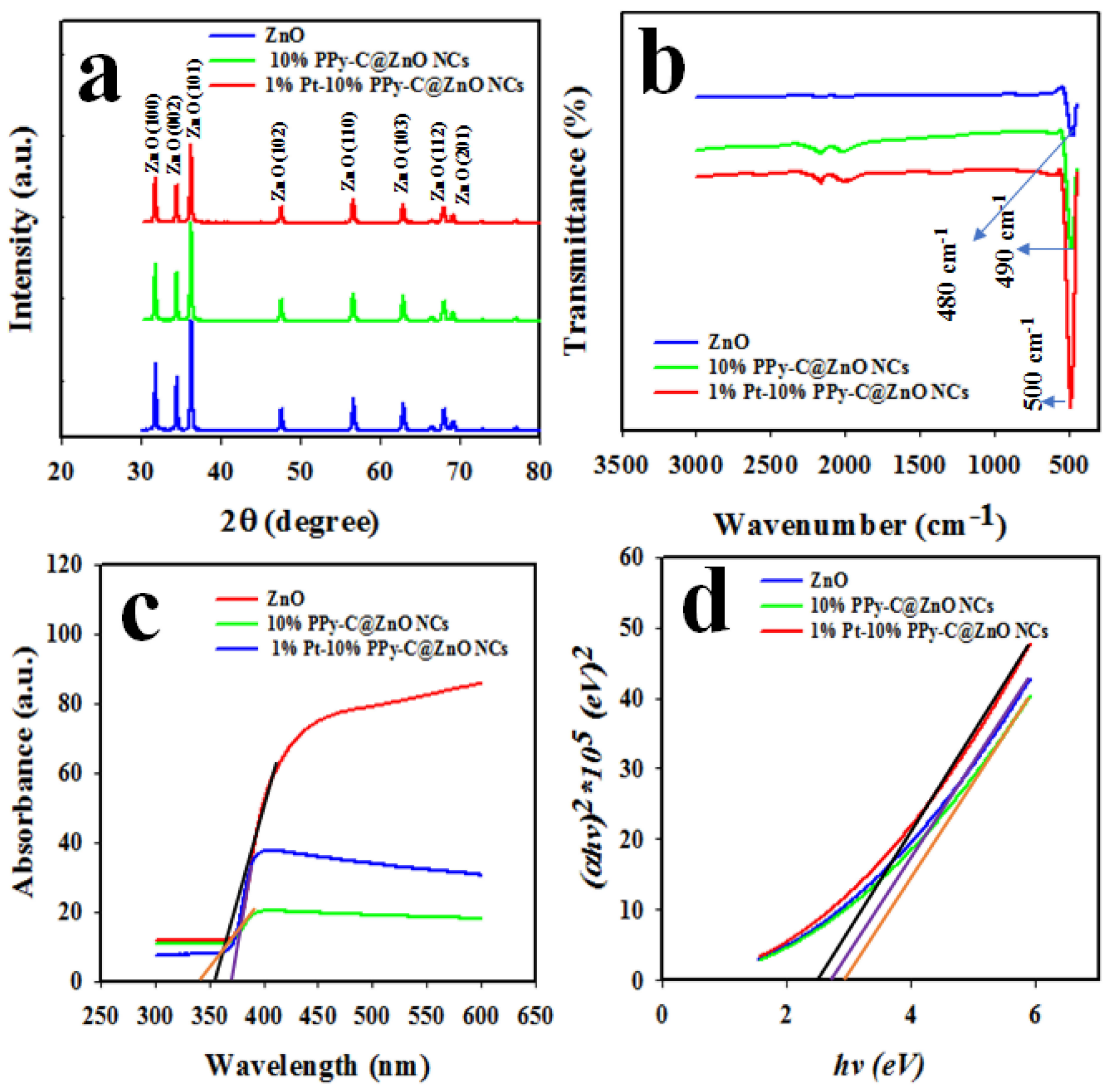
The structural and optical characterization of Pt-NP-embedded PPy-CB@ZnO NCs; (**a**) powder XRD pattern, (**b**) FTIR, (**c**) the UV-visible absorption by prepared NCs, and (**d**) Tauc’s plot using the UV-visible optical absorbance.

A crystallographic analysis of the synthesized NCs of the Pt-NP-embedded PPy-CB@ZnO NCs was performed, as illustrated in Figure 6a. The XRD pattern exhibits the crystalline planes of ZnO at (100), (002), (102), (110), (103), (112), and (201) only, also identified in previous reports [35,36]. The functional groups of the Pt-NP-embedded PPy-CB@ZnO NCs were subject to FTIR investigation, as illustrated in Figure 6b. 

A peak was obtained at 500 cm^−1^, identified as the stretching vibration of the Zn-O bond [37,38]. Using Tauc’s equation, the optical band-gap energy of the NCs was calculated from the UV–visible absorbance, as presented in Figure 6c,d. As measured, the optical band-gap energy of the Pt-NP-embedded PPy-CB@ZnO NCs is equal to 2.8 eV, which is less than the bandgap of the PPy-CB@ZnO NCs (3.2 eV) and ZnO (3.0 eV). Therefore, the prepared Pt-NP-embedded PPy-CB@ZnO NCs have more conductivity compared to PPy-CB@ZnO and ZnO.

### 3.4. Electrochemical Studies of Pt-NPs-Embedded PPy-CB@ZnO NCs

**Figure 7:**
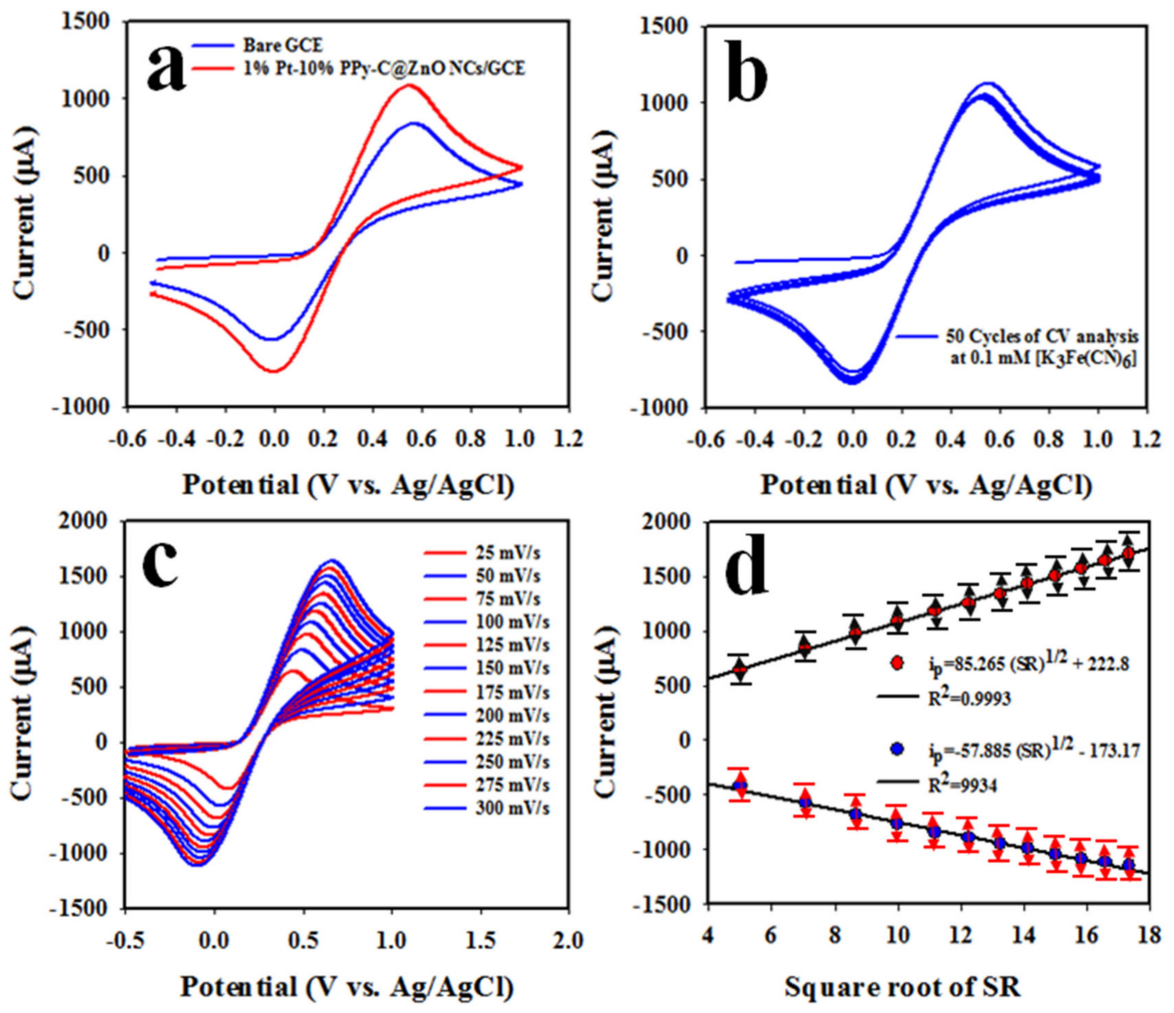
The electrochemical characterization of Pt−NPs- embedded PPy−CB@ZnO NCs/GCE by cyclic voltammetry; (**a**) The comparison of coated and bare−GCE in analysis of 0.1 mM K_4_[Fe(CN)_6_], (**b**) stability of coated GCE, (**c**) scan rate at 25~300 mV/s, and (**d**) current versus square root of SR.

For the voltammetric characterization of the prepared Pt-NP-embedded PPy-CB@ZnO NCs/GCE, 0.1 mM K_4_[Fe(CN)_6_] was used, as shown in Figure 7a. The separation potential between the oxidation and the reduction peak current of the coated GCE was +0.55 V. That of the bare GCE was +0.58 V, which is higher than that of the Pt-NP-embedded PPy-CB@ZnO NCs/GCE. Therefore, the working electrode based on the Pt-NP-embedded PPy-CB@ZnO NCs/GCE has more electric conductivity, favorable to the electrochemical analysis of an analyte. Similar observations have been described elsewhere [39,40]. Stability is an important sensor criterion in the electrochemical analysis of a working electrode. Thus, the stability of the Pt-NP-embedded PPy-CB@ZnO NCs/GCE was tested in the investigation of the 0.1 mM K_4_[Fe(CN)_6_], as presented in Figure 7b, and 50 CV cycles were completely indistinguishable. It is evident in the analysis of K_4_[Fe(CN)_6_] that the assembled working electrode has a high stability, and it can be assumed that other analytes will show a similar performance. To evaluate the molecular diffusion on the surface of the working electrode, a plot showing current versus the square root of the scan rate at a range of 25~300 mV/s is shown in Figure 7d. The experiment was conducted using 0.1 mM K_4_[Fe(CN)_6_]. The resulting peak currents are linearly distributed on the lines in both oxidation and reduction and can be expressed by the following equations (Equations (1) and (2)).

i_p_ = 85.265 (SR)^1/2^ + 222.8; R^2^ = 0.9993 in oxidation of K_4_[Fe(CN)_6_] (1)

i_p_ = 57.885 (SR)^1/2^ − 173.17; R^2^ = 0.9934 in reduction of K_3_[Fe(CN)_6_] (2)

**Figure 8:**
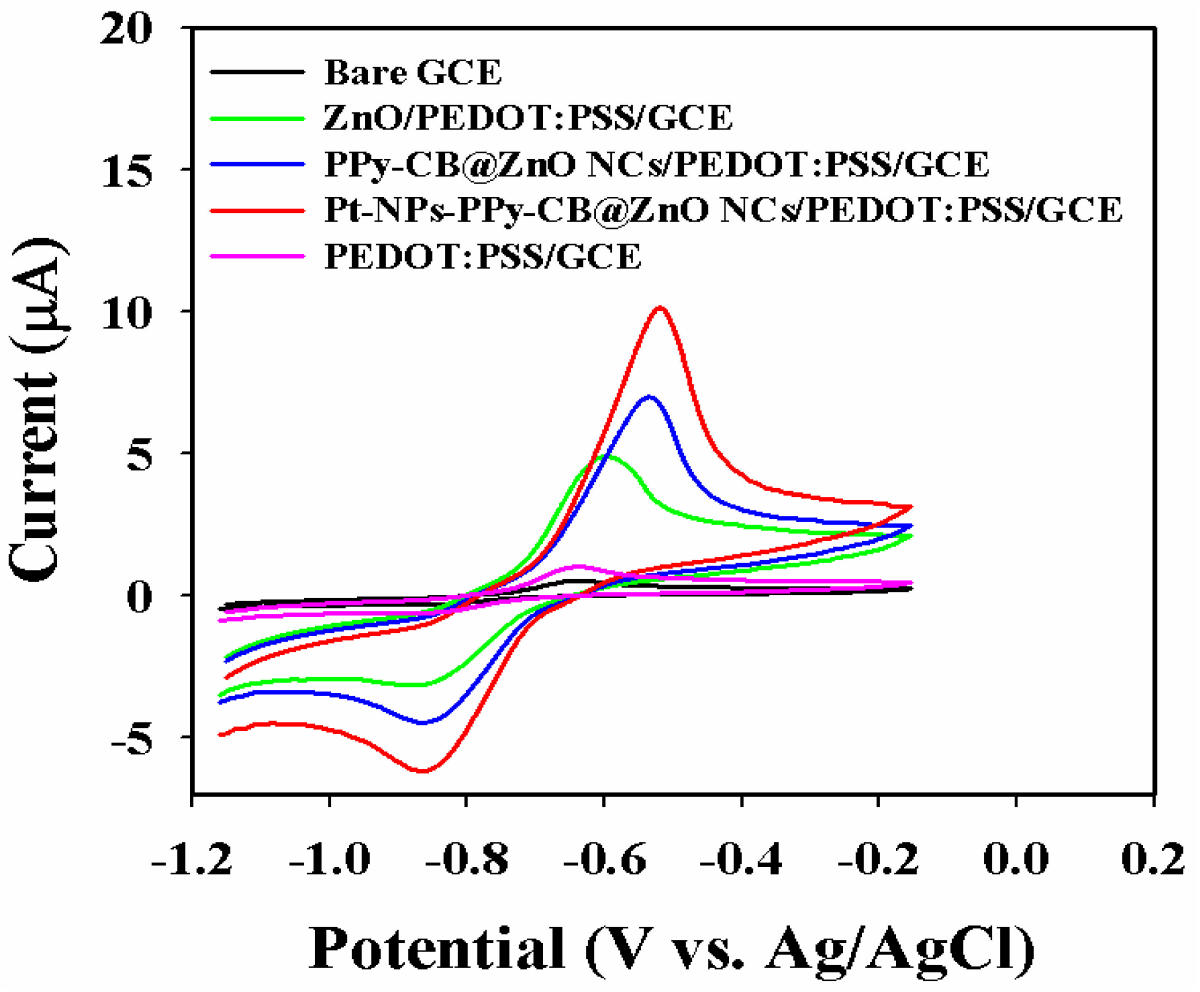
Control experiment executed at 16.5 µM of 4−NP using modifired GCE with various nanomaterials of constituting elements of Pt−NPs−embedded PPy−CB@ZnO NCs.

To justify the reactivity of Pt-NPs-embedded PPy-CB@ZnO NCs toward 4-NP, the constituting of each component of NCs were subjected to modify GCE individually and results are shown in Figure 8. As illustrated in Figure 8, Pt-NPs-embedded PPy-CB@ZnO NCs/GCE has the highest electrochemical reactivity toward 4-NP in 16.5 µM concentration in a phosphate buffer medium of pH 7.0 compared to only bare GCE, PEDPT:PSS/GCE, ZnO/PEDOT:PSS/GCE, and PPy-CB@ZnO NCs/PEDPT:PSS/GCE electrodes. To execute this experiment, cyclic voltammetry has been applied in the detection of 4-NP in room conditions in presence of different modified electrodes. Peak current is increased significantly with the Pt-NPs-embedded PPy-CB@ZnO NCs electrode in identical conditions compared to other electrode assembly due to attachment of electro-active species (Pt-NPs) onto surface PPy-CB@ZnO NCs. In this case, the large surface area of synthesized Pt-NPs-embedded PPy-CB@ZnO NCs is involved as a substantial factor for this enhancement of resultant current and peak separation in electro-catalytic performance of nanomaterials.

**Figure 9:**
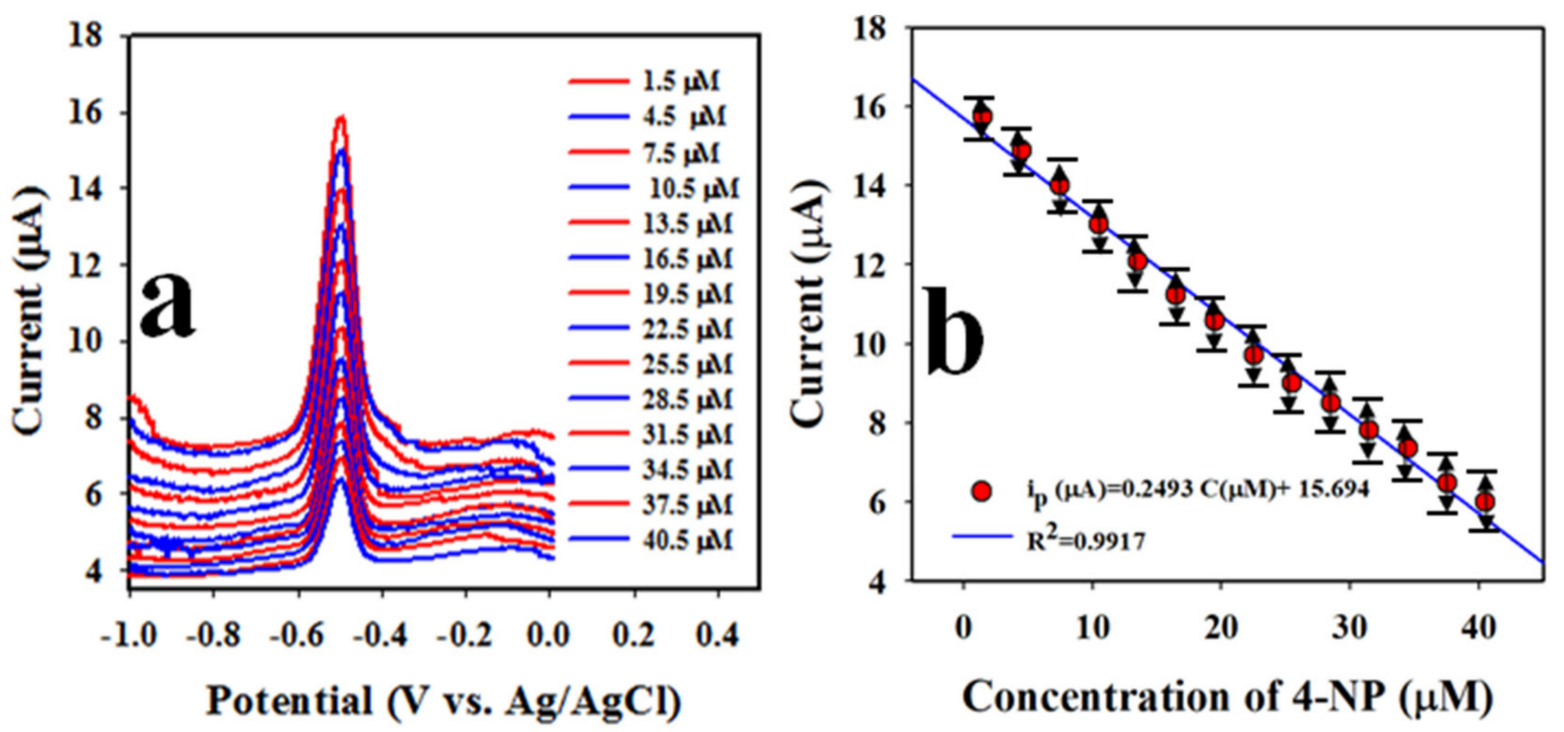
Detection of target analyte with Pt−NPs−embedded PPy−CB@ZnO NCs/GCE; (**a**) the resulted DPV analysis of 4−NP at 7.0 pH buffer and (**b**) calibration of 4−NP sensor obtained from current versus concentration of 4−NP.

The two equations above confirm good linearity and that the rate of reactions is diffusion-controlled rate of molecules on the working electrode surface, which has also been shown by previous authors [41]. In the current approach, differential pulse voltammetry (DPV) is a potential electrochemical method for analyzing an analyte. Therefore, 4-NP in a buffer medium of pH 7.0 was subject to DPV analysis based on a concentration range of 1.5~40.5 µM, as presented in Figure 9a. It is clear that the intensity of the peak current decreased (4-NP reduction held on −0.5 V) as the concentration of 4-NP increased. Thus, peak current points are plotted against the corresponding concentrations of 4-NP in Figure 9b. 

The obtained current is distributed in a linear manner at a 4-NP concentration range of 1.5~40.5 µM, which was denoted as the detection range (LDR) of 4-NP for the assembled electrochemical sensor. Sensor parameters such as sensitivity (7.8892 µAµM^−1^ cm^−2^) were calculated from the slope of the LDR (0.2493 µAµM^−1^) by considering the apparent surface area of the GCE (0.0316 cm^2^). The LOD is estimated as 1.25 ± 0.06 µM, which is calculated from 3xSD/σ (SD: Standard deviation of blank response; σ: Slope of the calibration curve). Limit of quantification (LOQ) is also calculated as 3.79 µM from LOQ = 10LOD/3.3. The linear equation of LDR can be expressed by Equation (3) as follows.

i_p_ = 0.2493 C(µM) + 15.694; R = 0.9917 in reduction of 4-NP (3)

**Figure 10:**
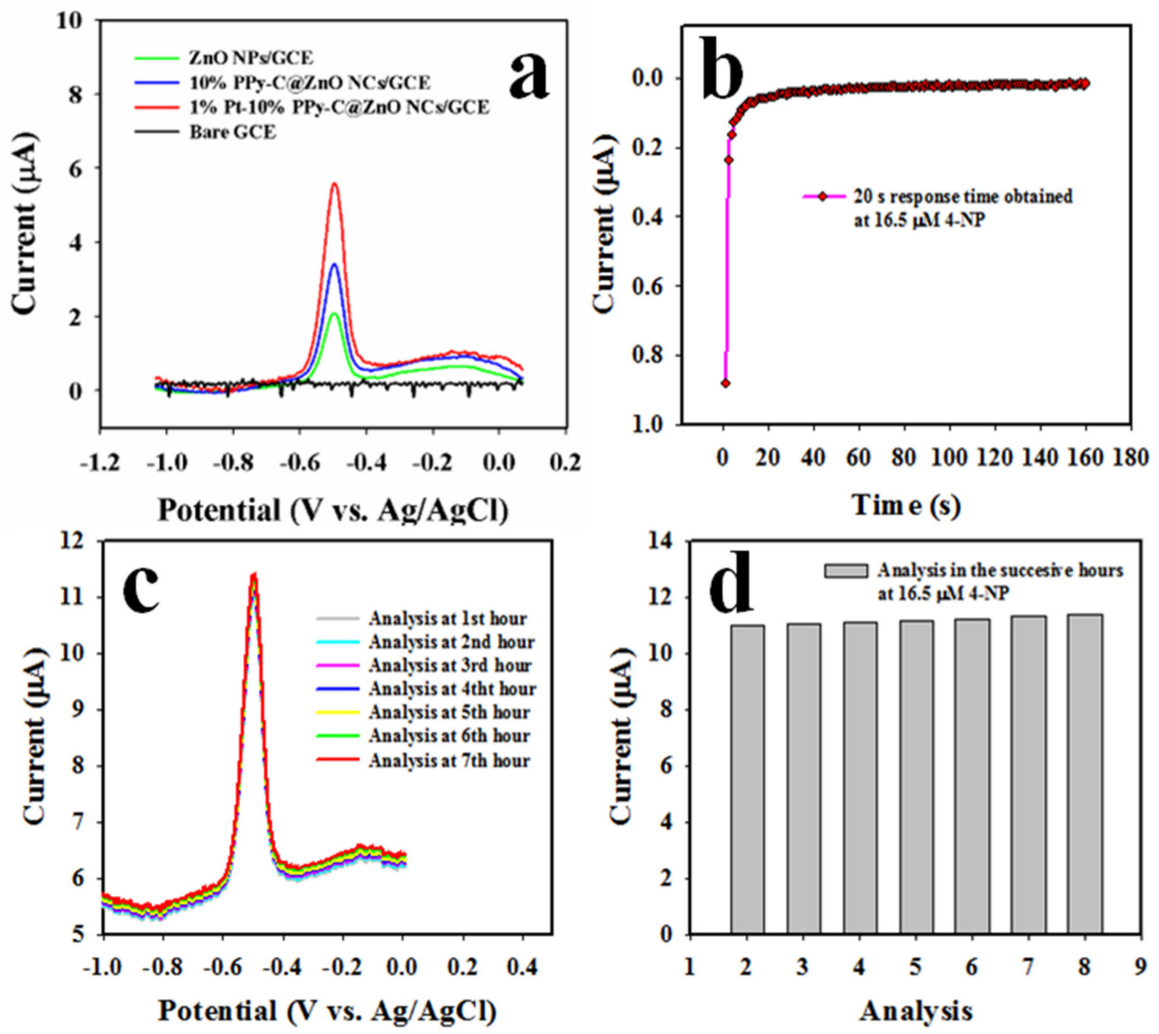
Analysis of sensor performance; (**a**) Control experiment in DPV analysis of 4−NP, (**b**) Response time at current versus time, (**c**) Reproducibility of 4−NP sensor, and (**d**) Reproducibility bar diagram.

A control experiment was performed, as illustrated in Figure 10a, to evaluate the reduction performances of 4-NP by the constituting elements of the prepared NCs, such as ZnO, PPy-CB@ZnO, and Pt-NPs on the PPy-CB@ZnO NC-coated GCE. 

The GCE modified by Pt-NPs-embedded PPy-CB@ZnO NCs has the highest peak current, and the bare GCE has a zero peak current. Thus, for 4-NP analysis, Pt-NPs-PPy-CB@ZnO NCs reasonably improved the conductivity of the working electrode via the DPV method. To specify the response of the 4-NP sensor, 16.5 µM of 4-NP was subjected to analysis, and the results are plotted as current versus time in Figure 10b. The current responses became steady at 20 s. Thus, 20 s was considered the response time of the 4-NP sensor for electrochemical analysis. The reproducibility of an electrochemical sensor is an important parameter that provides reliability information about it. Thus, the reproducibility test was executed by reaping seven analyses of 4-NP at 16.5 µM in a phosphate buffer medium of pH 7.0 using the sample working electrode, as shown in Figure 10c and a bare diagram in Figure 10d. As shown, the currents at the peak point in the reduction of 4-NP are changed and cannot be separated from each other. The related standard deviation was calculated and found as 1.28%. Thus, this test confirms that the assembled sensor based on the Pt-NPs-embedded PPy-CB@ZnO NCs/GCE has good reliability for analyzing 4-NP. 

**Figure 11:**
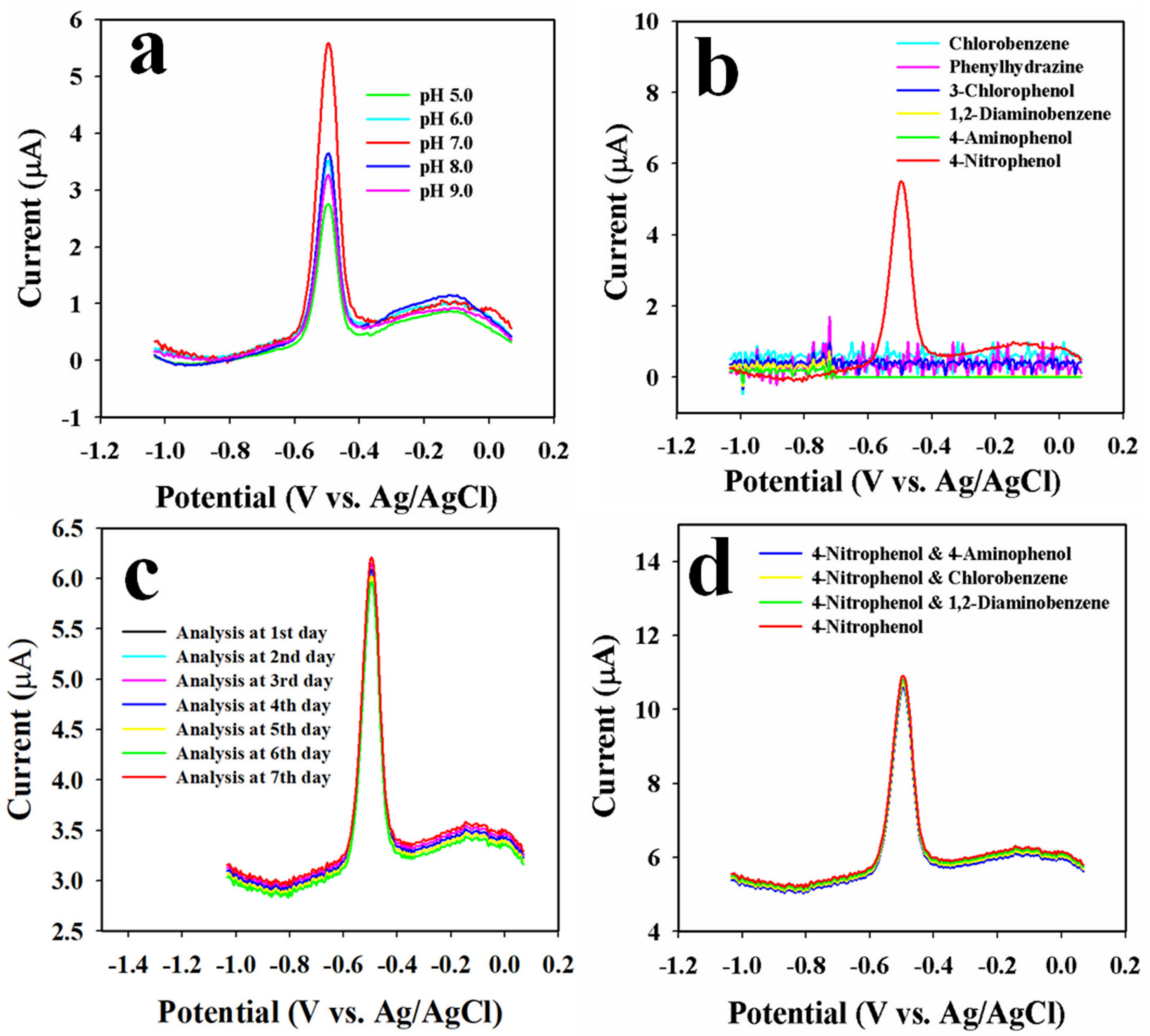
(**a**) The DPV investigation of 4−NP analysis at distinct pH value of buffer medium, (**b**) DPV analysis of various toxic chemicals in 16.5 µM concentration, (**c**) the repeatability performance of 4−NP sensor at 4.5 µM concentration of 4−NP I buffer medium of pH 7.0, and (**d**) interference effects of 4−NP sensor probe.

The pH value of the buffer medium is an influencing parameter to analyze the toxic chemical. Therefore, a number of buffer media with various pH values ranging from acidic to basic (pH 5.0 to pH 9.0) was subjected to analyze the response of 4-NP and are presented in the Figure 11a. As illustrated, the 4-NP sensor with Pt-NPs-embedded PPy-CB@ZnO NCs-sensing substrate shows the highest current at pH 7.0 compared to other pH values. Thus, pH 7.0 of phosphate buffer medium is optimized and used for measuring by the DPV analysis to detect 4-NP with Pt-NPs-embedded PPy-CB@ZnO NCs/GCE electrode in room conditions. Selectivity is another significant parameter of any newly fabricated sensor probe to show in which chemical it is more efficient in detecting the target analyte. Therefore, a number of toxic chemicals including chlorobenzene (CB), phenyl-hydrazine (Phyd), 3-chlorophenol (3-CP), 1,2-diaminobenzene (1,2-DAB), 4-aminophenol (4-AP), and 4-nitrophenol (4-NP) were taken to DPV analysis with GCE-modified Pt-NPs-embedded PPy-CB@ZnO NCs. The results are presented in Figure 11b. As shown, for 4-NP it exhibited the highest peak current (magnitude) compared to other chemicals in similar measurements in identical conditions. Other chemicals are not found to have any enhanced current response towards the sensing substrate (Pt-NPs- embedded PPy-CB@ZnO NCs) in 7.0 pH buffer medium. It can be concluded that Pt-NPs-embedded PPy-CB@ZnO NCs/GCE electrode has detected selectivity of 4-NP, only compared to other interferences.

To realize the stability of Pt-NPs embedded PPy-CB@ZnO NCs/GCE sensor probe, the repeatability performance was tested again but for elongated period around seven consecutive days, as illustrated in Figure 11c. A similar observation is perceived as in the Figure 10c. This experiment was performed at 4.5 µM of 4-NP in a buffer medium of pH 7.0. Thus, it can be predicted that the 4-NP sensor based on Pt-NPs-embedded PPy-CB@ZnO NCs/GCE is stable in its performance. To investigate the interference of 4-NP sensor probe, the 4-NP was analyzed by DPV in presence of CB, 4-AP, 1,2-DAB and CB, as illustrated in Figure 11d. It is perceived that the 4-NP sensor probe has not any measurable interference in presence of other toxic chemicals.

Comparison studies of 4-NP sensors based on various sensing electrode materials are demonstrated [42,43,44,45,46,47,48,49,50,51] in Table 1.

**Scheme 2:**
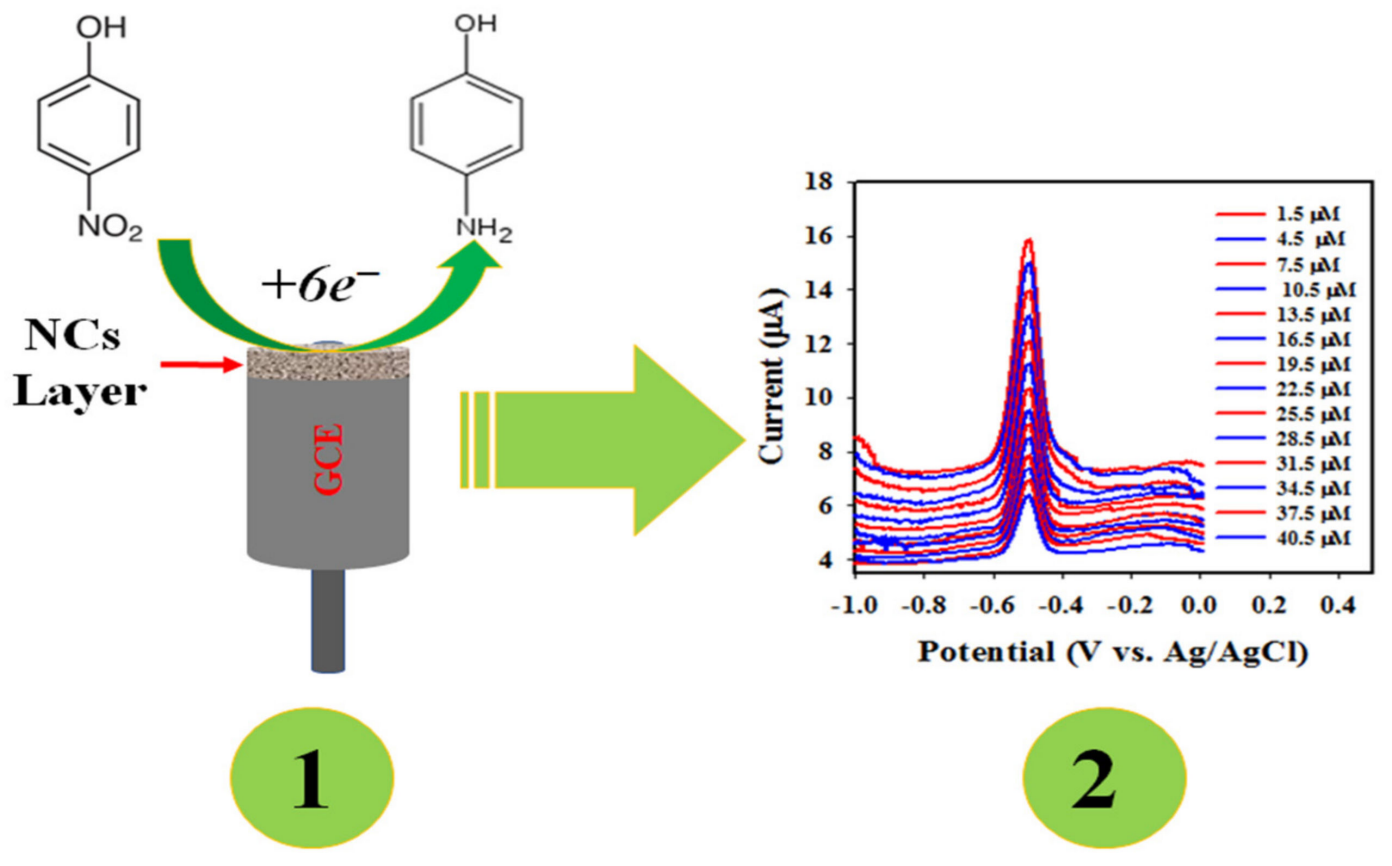
Schematic representation of chemical sensing; (**1**) electrochemical reduction of 4−NP and (**2**) the concentration-based responses of 4−NP to 4−AP.

As shown in Table 1, the 4-NP sensor based on the PEDOT:PSS/GCE modified by Pt NPs-embedded PPy-CB@ZnO NCs exhibited improved performances in terms of sensitivity, LOD, and LDR. The electrochemical quantification of 4-NP based on Pt NPs-embedded PPy-CB@ZnO NCs/GCE is presented in Scheme 2. As shown, 4-NP is adsorbed on the surface of the modified GCE. Due to the applied potential, it is reduced to 4-aminophenol, as indicated in the reaction scheme below. Comparable electrochemical reduction reactions of 4-NP are reported in other articles [52,53,54]. The suggested electrochemical reaction of 4-NP on the Pt NPs-embedded PPy-CB@ZnO NCs/GCE surface is as follows (Equations (4)–(6)):

HO-C_6_H_4_-NO_2_ + 2e^−^ → HO-C_6_H_4_-NO + H_2_O (4)

HO-C_6_H_4_-NO + 2e^−^ → HO-C_6_H_4_-NHOH (5)

HO-C_6_H_4_-NHOH + 2e^−^ → HO-C_6_H_4_-NH_2_ + H_2_O (6)

Finally, the Pt-NP-embedded PPy-CB@ZnO NCs/PEDOT:PSS/GCE-fabricated sensor probe was used for the sensor validation by electrochemical analysis with real environmental samples, which were collected from various environmental sources, including underground water, tap water, and sea water. This also facilitated validation of the feasibility of 4-NP in environmental collected water samples. No electrochemical response of 4-NP was found in all the samples, which may be due to the absence of 4-NP in the water samples or because the concentration of 4-NP was lower than the method’s detection limit. Therefore, this validity analysis was performed by applying the recovery standard addition method in room conditions. For validity, the real water substrates are spiked with 4-NP. The spiked concentration of 4-NP was measured based on the calibration curve, which is shown in Figure 9b. The data of real sample analyses are presented in Table 2. The 4-NP sensor based on the Pt-NP-embedded PPy-CB@ZnO NCs/PEDOT:PSS/GCE is shown to be reliable, with acceptable results [55,56,57,58,59,60,61,62,63,64]. Thus, the sensor can potentially be used for the development of micro-sized electrochemical devices by using an electrochemical approach. 

## 4. Conclusions

In this approach, the Sol–gel prepared nanocomposites (NCs) of Pt-NP-embedded polypyrrol carbon black-embedded ZnO (PPy-CB@ZnO) show a highly significant structural morphology, which is favorable to the electrochemical detection of 4-NP in a phosphate buffer medium at pH 7.0. A working electrode was fabricated with the help of a conducting coating binder (PEDOT:PSS) to stick the NCs onto the surface of the sensor probe. The fabricated sensor probe based on the Pt-NP-embedded PPy-CB@ZnO NCs/PEDOT:PSS/GCE was used for the detection of 4-NP in a wider range of 1.5~40.5 µM by using PEDOT:PSS. Besides this, the fabricated sensor probe exhibited the highest sensitivity (7.8892 µAµM^−1^ cm^−2^) and a relatively low LOD (1.25 ± 0.06 µM) and LOQ (3.79 µM). In addition, it has good reproducibility as well as a short response time. The 4-NP sensor was applied to analyze a real sample and exhibited significant and reliable performance. Thus, it introduced an easy-to-use approach in using an electrochemical technique for developing a nanocomposite-based sensor probe, and it has potential environmental applications for the safety of environmental fields on a broad scale. 

## Figures and Tables

**Table 1:** Comparison of sensor performances with various modified electrode towards the detection of 4-NP by an electrochemical approach.

Electrode Materials	LOD	LDR	Sensitivity	Ref.
PCZ/N-GE/GCE	0.062 μM	8 × 10^−7^~2 × 10^−5^ M	---	[42]
S-GCN NSs	0.0016 μM	0.05~90 μM	---	[43]
NiO/CeO_2_ NCs/GCE	2.48 μM	1~20 μM	3.68 AμM^−1^ cm^−2^	[44]
BaSnO_3_-gC_3_N_4_ NCs/GCE	1.0 μM	1.6~50 μM	0.81 μAμM^−1^ cm^−2^	[45]
Au-NP/RGO NCs/GCE	0.01 μM	0.05~2.0 μM	---	[46]
Au/CaCO_3_ NCs	0. 54 nM	0.1~100 μM	---	[47]
PDDA-G NCs/GCE	0.02 μM	0.06~110 μM	---	[48]
RGO-MIP NCs	0.005 μM	0.01~100.0 μM	---	[49]
GO/GCE	0.02 μM	0.1~120 μM	---	[50]
Ag-NWs-PANI NCs/GCE	52.0 nM	0.6~32 μM	---	[51]
Pt NPs-PPy-CB@ZnO NCs/GCE	12.50 nM	1.5~40.5 µM	7.89 μAμM^−1^ cm^−2^	This study

**Table 2:** Validity analysis of environmental real-samples with Pt-NPs-embedded PPy-CB@ZnO NCs/PEDOT:PSS/GCE sensor probe, using an electrochemical approach.

Real Samples	Added 4-NP Conc. (µM)	Found 4-NP Conc. ^a^ (µM)			Average Recovery ^b^ (%)	RSD ^c^ (%) (*n* = 3)
		R1	R2	R3		
Underground water	16.50	16.46	16.37	16.42	99.49	0.27
	7.20	7.05	7.08	6.89	97.31	0.34
Sea water	16.50	16.27	16.35	16.47	99.17	0.62
	7.20	6.92	7.04	6.99	96.99	0.39
Tap water	16.50	16.21	16.15	16.19	98.08	0.19
	7.20	7.03	7.08	6.97	96.30	0.36

^a^ Mean of three repeated determination with Pt-NP-embedded PPy-C@ZnO NCs/PEDOT:PSS/GCE. ^b^ Concentration of 4-NP determined/Concentration taken. (Unit: µM). ^c^ Relative standard deviation value indicates precision among three repeated measurements (R1, R2, & R3).

## Data Availability

Data will be available upon reasonable request.
